# Single‐cell transcriptome atlas identified novel regulators for pigment gland morphogenesis in cotton

**DOI:** 10.1111/pbi.14035

**Published:** 2023-03-10

**Authors:** Lu Long, Fu‐Chun Xu, Chun‐Hu Wang, Xiao‐Tong Zhao, Man Yuan, Chun‐Peng Song, Wei Gao

**Affiliations:** ^1^ State Key Laboratory of Cotton Biology; School of Life Science Henan University Kaifeng China; ^2^ State Key Laboratory of Crop Stress Adaptation and Improvement Henan University Kaifeng China

**Keywords:** *Gossypium*, cell differentiation, specialized structure, transcription factors, terpenoids

Cotton (*Gossypium* spp.) is a leading economic crop that is grown in more than 50 countries. The cottonseeds, once regarded as the by‐product of fibre production, contain a rich supply of unsaturated fatty acids, proteins and vitamins. To date, the annual production of cottonseeds has the potential to meet the protein requirements for 550 million people globally, which shows great potential as a food resource amidst a growing food shortage (Janga *et al*., 2019). However, the utilization of cottonseed for food purposes is limited owing to the presence of ‘pigment glands’, which contains gossypol and its derivatives that are toxic to humans (Gao *et al*., 2020).

To study how pigment gland cells differentiate and to reveal the gene regulatory network in gland morphogenesis, scRNA‐Seq was performed using a pair of NILs (gland cotton ‘CCRI12’ and glandless cotton ‘CCRI12gl’). The 1‐week‐old cotyledons were enzymatically digested, and the purified protoplasts were labelled with a 10x genomics barcode for high‐throughput sequencing (Figure 1a). A total of 9186 individual cells, including 4790 cells from ‘CCRI12’ and 4396 cells from ‘CCRI12gl’, were obtained after cell filtering process (Figure S1, Table S1) and were divided into 12 clusters based on highly variable genes (Figure 1b, Figure S2).

**Figure 1:**
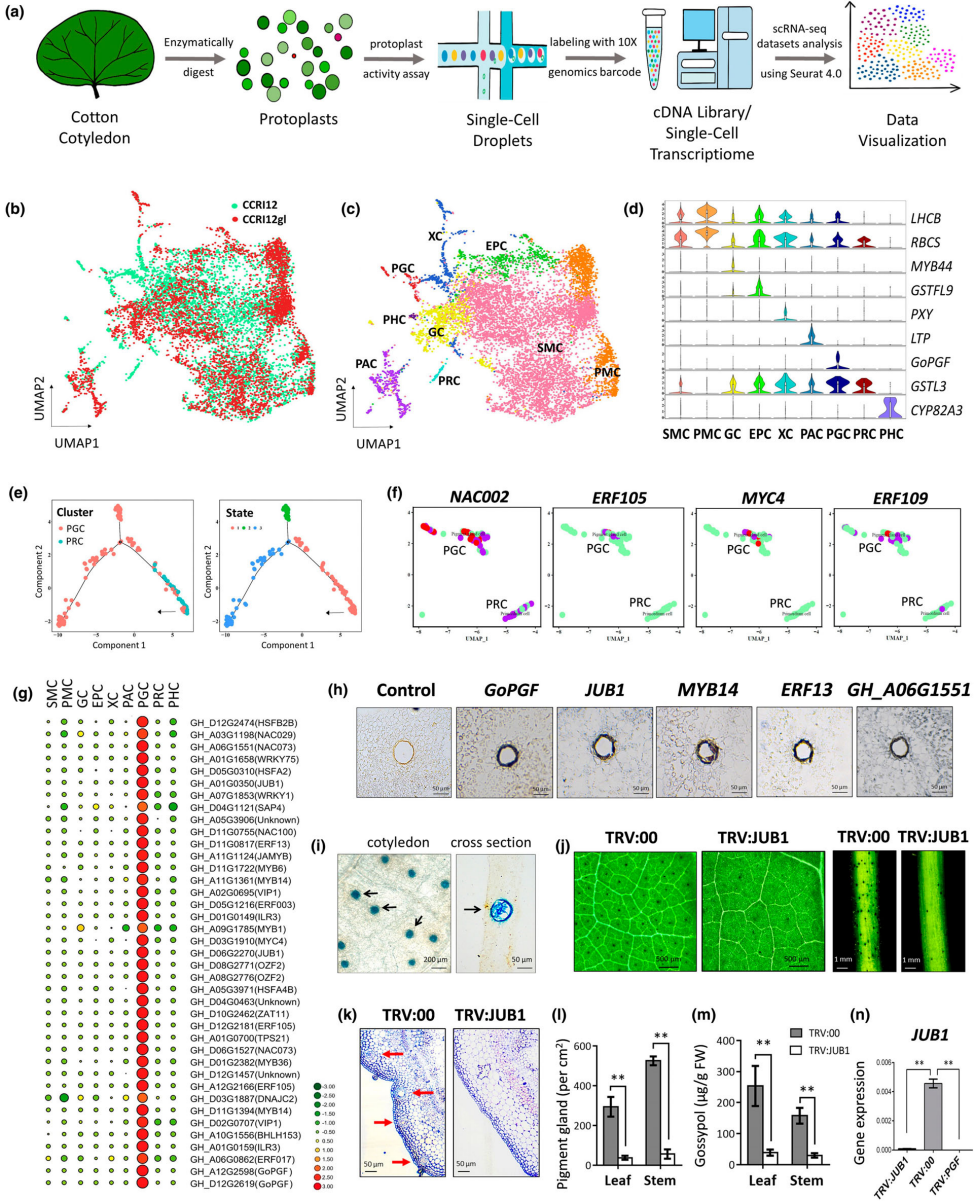
Development and application of scRNA‐Seq on cotton cotyledons. (a) General workflow for signal‐cell sequencing of the cotton cotyledons. (b) Distribution of individual cells is shown with a UMAP map. (c) The UMAP map shows the corrected classification of nine cell clusters. (d) Violin plots show the expression of the distribution of marker genes in different cell types. (e) Distribution of PGC and PRC along with cell clusters and branch states. (f) The distribution of expression of representative TFs in PRC and PGC. (g) Dot plots that show the expression of representative TFs in different cell types. (h) RNA *in situ* hybridization of representative TFs in cotton cotyledon. (i) GUS staining of the cotyledon of Pro_GoPGF_‐GUS transgenic cotton. The black arrow indicates pigment glands. (j) The phenotype of *GhJUB1*‐silencing cotton. TRV:00, empty vector; TRV:JUB1, *GhJUB1*‐silenced cotton. (k) Observation of the gland structure on TRV:00 and TRV:JUB1 stems through transverse sections. (l) The number of visible pigment glands (*n* ≥ 10). (m) An LC‐ESI‐MS/MS analysis of gossypol contained (*n* ≥ 6). (n) *GhJUB1* expression in the leaves of TRV:00, TRV:JUB1, and TRV:PGF seedlings (*n* ≥ 4). Statistical analysis: **P < 0.01, *t*‐test.

To verify and correct cell group classifications, the expression profile of reported marker genes in 12 cell clusters of cotton cotyledons was studied. The clusters 0, 1 and 4 were identified as spongy mesophyll cells (SMC) due to the enrichment of a photosynthesis‐related gene *LHCB*, and clusters 3 and 6 were identified as palisade mesophyll cells (PMC) due to the high expression of *RBCS*. The dominantly expressed *GSTF9* marked cluster 5 as epidermal cells (EPC), and *GSTL3* identified cluster 10 as the primordial cells (PRC) that could differentiate. In addition, cell type that specifically expressed *MYB44*, *PXY*, *LTP* and *CYP82A3* marked the clusters 2, 7, 8 and 11 as guard cells (GC), xylem cells (XC), parenchyma cells (PAC) and phloem cells (PHC), respectively (Figures 1c,d, Table S2).

No well‐known marker gene for pigment gland cells has been reported to date. GoPGF is the key factor that controls the biogenesis of pigment glands (Ma *et al*., 2016). However, the expression of *GoPGF* in different cell types has not been studied. Cluster 9 was identified in the cotyledons of gland cotton ‘CCRI12’ but not glandless cotton ‘CCRI12gl’, and *GoPGF* was specifically detected in the cells of cluster 9. This led to the tentative annotation of cluster 9 of the cotyledon cells as pigment gland cells (PGC; Figure 1c).

A pseudotime analysis was performed to uncover the differentiation relationships of cotyledon cell types. The study of the individual cell distribution and trajectory revealed that the PRCs originated earlier than the PGCs, suggesting that the PGCs could have differentiated from the PRCs (Figure 1e, Figure S3). In addition, four representative genes were selected to show their distribution and expression levels in PRCs and PGCs (Figure 1f).

To explore the potential regulators of gland development of cotton, the highly expressed genes in each cell cluster were studied. A total of 9325 DEGs were obtained with 1430 DEGs preferentially expressed in PGC, while the other cell clusters contained a range from 572 to 1704 (Table S3). Other than *GoPGF*, the previously reported *GhERF105* (Wu *et al*., 2021), which determines the biogenesis of pigment glands in cotton leaves, was also identified as PGC‐specific gene in our scRNA‐Seq data. These results suggested the reliability of scRNA‐Seq analyses in pigment gland cells and confirmed the accuracy of our classification of cell types.

To date, the regulators involving in pigment gland biogenesis that have been identified are all TFs, including CGF1, CGF2, GoPGF/CGF3, GaGRAS/GoSPGF and GhERF105. Therefore, this study focused on the TFs that were preferentially expressed in PGCs (Figure 1g, Table S4). qPCR revealed that most of the identified TFs were highly expressed in gland cells, while they were expressed at very low levels in the mesophyll cells (Figure S4). In addition, five candidate genes, including *GoPGF*, were selected for RNA *in situ* hybridization. These results showed that these genes have strong hybridization signals in the glandular structure (Figure 1h).

A 1.5‐kb promoter upstream of the *GoPGF* initiation codon was cloned to drive the expression of *GUS* in the cotton gland cultivar ‘Coker312’. The transgenic lines that expressed *GUS* were obtained and used for histochemical staining. As shown in Figure 1i, a strong and clear GUS staining was restricted to the pigment glands. To our knowledge, this study is the first to use GUS staining to demonstrate that the transcription of *GoPGF* is restricted to gland cells.

Virus‐induced gene silencing was utilized to quickly screen the candidate genes that controlled the formation of pigment glands. The results suggested that knock down of some candidate genes, including *ERF13* and *MYB14*, mildly reduced the gland density (Figure S5). Notably, the *GH_A05G3906* could modulate the contents of gossypol without changing the number of pigment glands, which suggested a possible biosynthetic pathway of sesquiterpene metabolism that is independent of pigment gland biogenesis (Figure S5). Among all the candidates, *JUNGBRUNNEN 1* (*GhJUB1*) is of particular interest. Knock down of *GhJUB1* inhibits gland biogenesis and the accumulation of gossypol.

The *GhJUB1*‐silenced plants (TRV:JUB1) exhibited dramatically reduced pigment glands in newly growing tissues (Figure 1j–l). In addition, *GhJUB1*‐silenced cotton plants exhibited gossypol levels of 15% in the leaves and 18% in the stems compared with those of the control plants (Figure 1m). These results revealed that *GhJUB1* regulates gland morphogenesis, which was similar to that of GoPGF. To study the relation between *GhJUB1* and *GoPGF*, the expression of *GhJUB1* was studied in the *GoPGF*‐silenced cotton (TRV:PGF), and the results showed that the expression of *GhJUB1* dramatically decreased to an undetectable level (Figure 1n), which suggests that GhJUB1 could be downstream of GoPGF to control the biogenesis of pigment glands.

The pigment gland of cotton is a highly distinctive structure, which provides an ideal system to study cell differentiation and organogenesis. Our study indicates that the initiation of cell differentiation of pigment glands is highly correlated with the specific expression of key genes. One of the major constraints in the study of glandular development of cotton is the lack of natural glandless mutants. The scRNA‐Seq data that we provide is invaluable for producing novel glandless mutants, which will greatly accelerate the breeding of commercially desired cotton varieties with glandless seeds.

## Conflict of interest

The authors declare no competing financial interest.

## Author contributions

W.G. and L.L. wrote the paper. F.C.X., C.H.W., M.Y. and X.T.Z. performed the experiments. C.P.S. designed the experiments. All authors approved the final manuscript.

## Supporting information


**Figure S1** Distribution of the medium number of gene and UMI of each sample before filtered (a) and after filtered (b).
**Figure S2** UMAP visualization of the cell clusters of ‘CCRI12’ and ‘CCRI12gl’.
**Figure S3** Pseudotime trajectory analysis identified the trajectory map of gland cells.
**Figure S4** PGC‐specific expression of representative TFs in PGC and MC of cotton cotyledon was analysed with qPCR.
**Figure S5** Functional analysis of TFs specifically expressed in PGCs by VIGS.

**Table S1** Numbers and median UMI of captured cells.
**Table S2** Cell number and DEGs of major cell clusters in cotton cotyledon.
**Table S3** All identified DEGs in different cell cluster.
**Table S4** Gland‐specific transcription factors in PGC cluster.
