# Supplementary material for: Single‐cell transcriptome atlas identified novel regulators for pigment gland morphogenesis in cotton
Source: Plant Biotechnol J. 2023 Mar 10;21(6):1100–2. doi: 10.1111/pbi.14035 (PMC10214745; doi:10.1111/pbi.14035)
Supplement: Supplementary file 1 — Figure S1 Distribution of the medium number of gene and UMI of each sample before filtered (a) and after filtered (b). Figure S2 UMAP visualization of the cell clusters of ‘CCRI12’ and ‘CCRI12gl’. Figure S3 Pseudotime trajectory analysis identified the trajectory map of gland cells. Figure S4 PGC‐specific expression of representative TFs in PGC and MC of cotton cotyledon was analysed with qPCR. Figure S5 Functional analysis of TFs specifically expressed in PGCs by VIGS. [file PBI-21-1100-s002.docx]

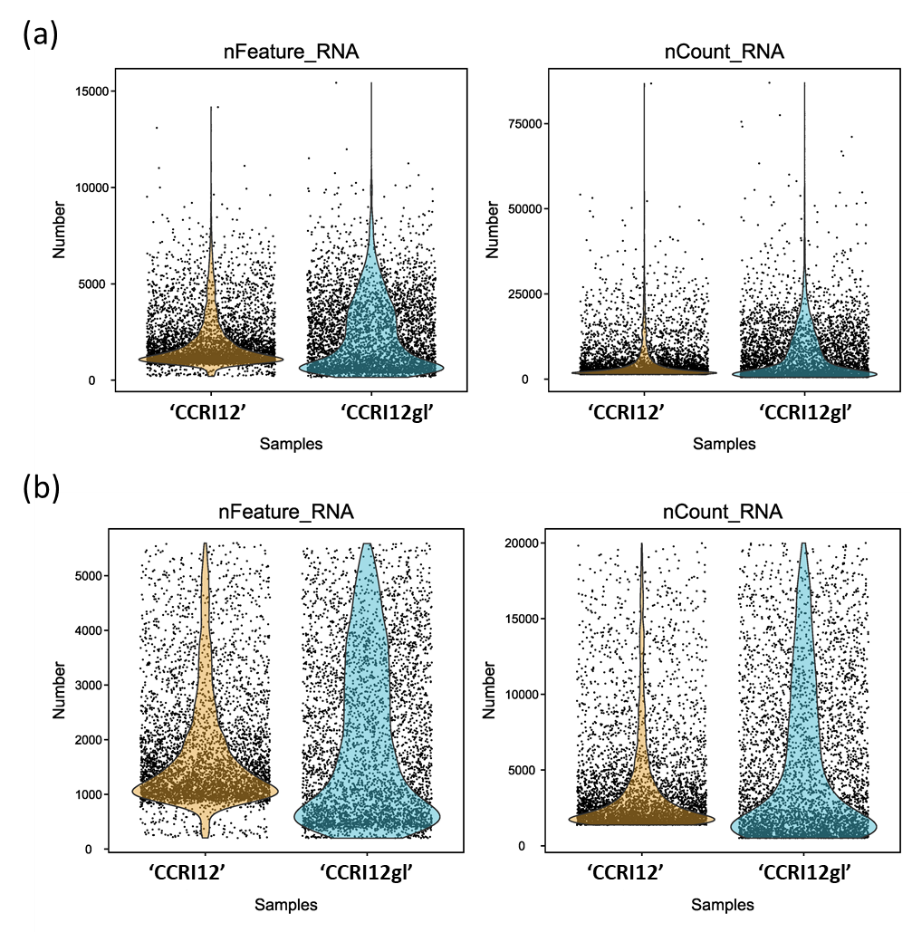


Figure S1. Distribution of the medium number of gene and UMI of each sample before filtered (a) and after filtered (b).


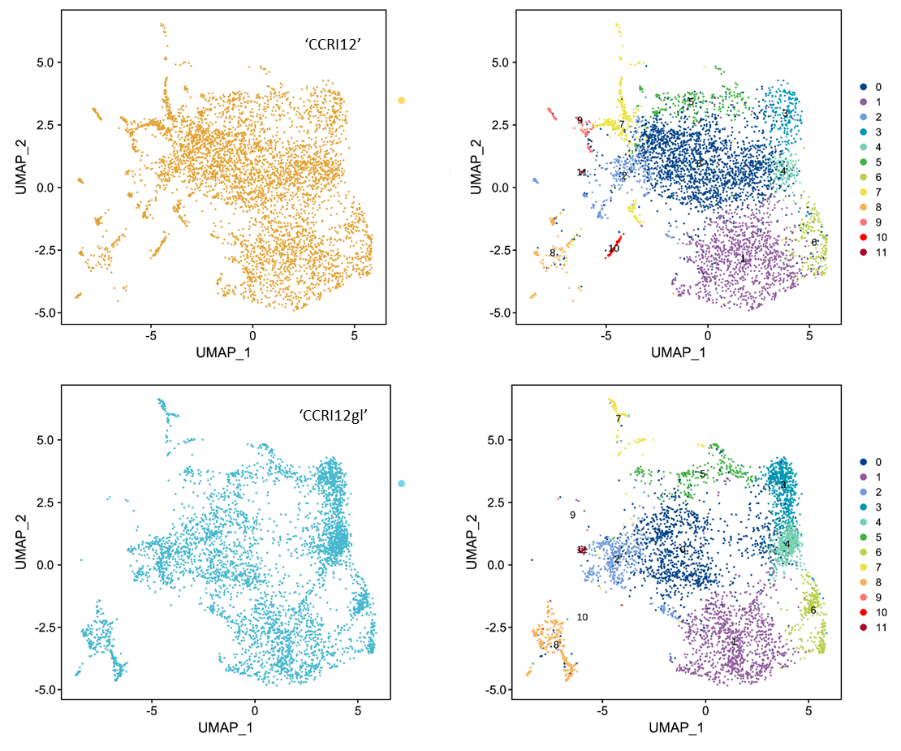


Figure S2. UMAP visualization of the cell clusters of ‘CCRI12’ and ‘CCRI12gl’.


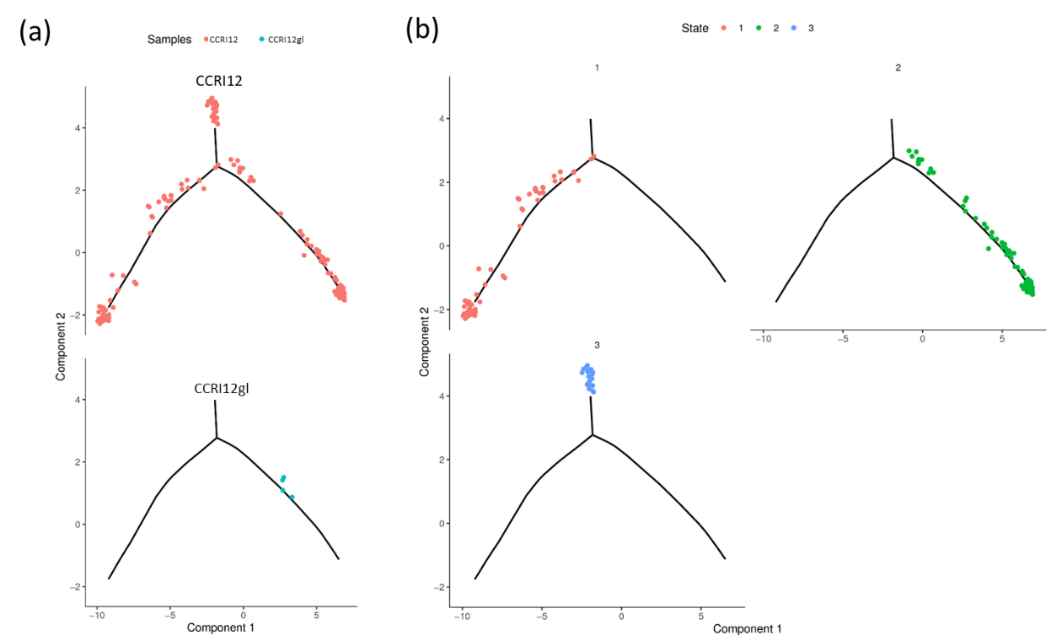


Figure S3. Pseudotime trajectory analysis identified the trajectory map of gland cells. (a) The distribution of ‘CCRI12’ and ‘CCRI12gl’ cotton cultivar cells in a trajectory map. (b) Three state profiles of cell differentiation based on the trajectory analysis.


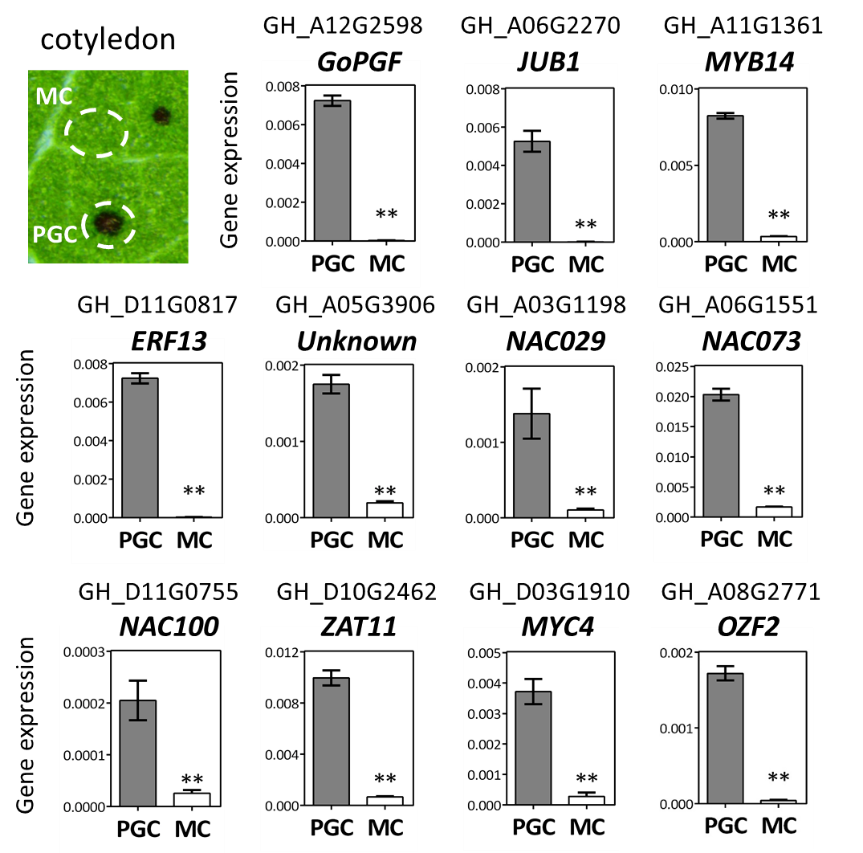


Figure S4. The PGC-specific expression of representative TFs in PGC and MC of cotton cotyledon was analyzed with qPCR. The *GhUB7* (*ubiquitin 7*, Accession: DQ116441) gene was used as an internal control (*n* ≥ 4, ***P* < 0.01, *t*-test).


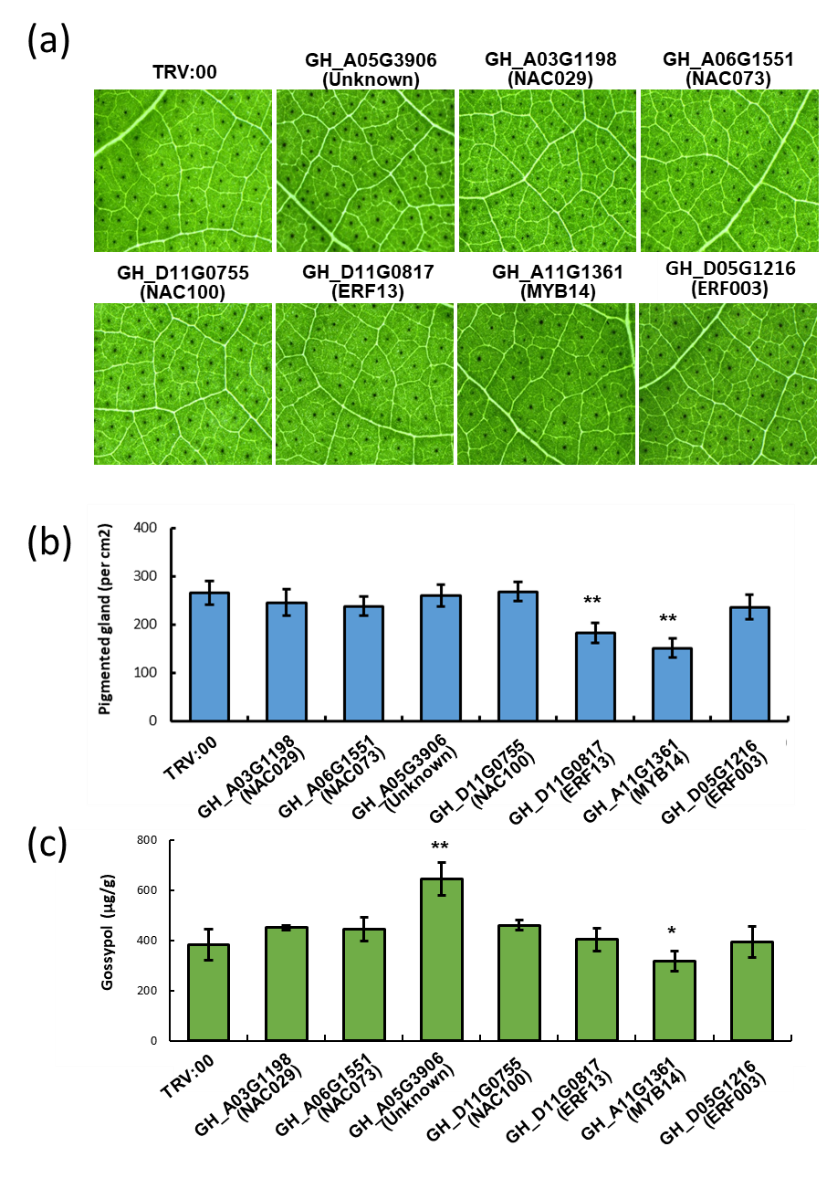


Figure S5. Functional analysis of TFs specifically expressed in PGCs by VIGS. (a) The phenotype of representative TFs in glandular development by VIGS analysis. (b) The pigment gland number of the silenced plants compared to TRV:00. (c) The gossypol content measurement of the silenced plants compared to TRV:00.
